# Presencia de integrones y su correlación con la multirresistencia en *Salmonella entérica* serovar Typhimurium: revisión sistemática exploratoria

**DOI:** 10.7705/biomedica.6816

**Published:** 2024-05-30

**Authors:** Nancy Yaneth Flórez, Claudia Silva, José Miguel Villarreal, Magdalena Wiesner

**Affiliations:** 1 Grupo de Microbiología, Dirección de Investigación en Salud Pública, Instituto Nacional de Salud, Bogotá, D. C., Colombia Grupo de Microbiología Instituto Nacional de Salud Bogotá D. C Colombia; 2 Doctorado en Ciencias de la Salud, Facultad de Medicina, Universidad Antonio Nariño, Bogotá, D. C., Colombia Universidad Antonio Nariño Facultad de Medicina Universidad Antonio Nariño Bogotá D. C Colombia; 3 Instituto de Biotecnología, Universidad Nacional Autónoma de México, Cuernavaca, México Universidad Nacional Autónoma de México Universidad Nacional Autónoma de México Cuernavaca Mexico; 4 Grupo de Bioquímica y Biología Molecular de las Micobacterias, Facultad de Ciencias, Universidad Nacional de Colombia, Bogotá, D. C., Colombia Universidad Nacional de Colombia Facultad de Ciencias Universidad Nacional de Colombia Bogotá D. C Colombia; 5 Grupo de Investigación en Enfermedades Infecciosas, Facultad de Medicina, Universidad Nacional de Colombia, Bogotá, D. C., Colombia Universidad Nacional de Colombia Facultad de Medicina Universidad Nacional de Colombia Bogotá D. C Colombia

**Keywords:** *Salmonella* Typhimurium, resistencia a múltiples medicamentos, islas genómicas, integrones, salud pública, *Salmonella* Typhimurium, drug resistance, multiple, genomic islands, integrons, public health

## Abstract

La multirresistencia a los antibióticos en *Salmonella entérica* serovar Typhimurium (Typhimurium) se asocia con integrones que portan genes de resistencia y que son dispersados por elementos genéticos móviles.

En esta revisión sistemática exploratoria, se buscó identificar los tipos de integrones y sus genes de resistencia en aislamientos de Typhimurium multirresistentes a antibióticos. Se realizó una búsqueda de artículos en Medline, PubMed, SciELO, ScienceDirect, Redalyc y Google Académico, publicados entre el 2012 y el 2020, en español o inglés, con las palabras claves: "integrons", "antibiotic resistance" y *"Salmonella* Typhimurium". En el análisis se incluyeron 38 artículos que reportaron multirresistencia a cinco familias de antibióticos. Los integrones de clase 1 con casetes de genes *aadA2* y bla_PSE-1_ fueron los predominantes, algunos probablemente relacionados con la isla genómica de *Salmonella* 1. No se encontraron integrones de clase 1 y 2 en un mismo aislamiento, ni se reportaron integrones de clase 3. La presencia de integrones explica en gran medida los perfiles de resistencia encontrados en aislamientos de diferentes fuentes de 15 países.

## Abstract (en)

In *Salmonella enterica* serovar Typhimurium (Typhimurium), multidrug resistance is associated with integrons carrying resistance genes dispersed by mobile genetic elements. This exploratory systematic review sought to identify integron types and their resistance genes in multidrug resistance Typhimurium isolates. We used Medline, PubMed, SciELO, ScienceDirect, Redalyc, and Google Scholar as motor searchers for articles in Spanish or English published between 2012 and 2020, including the keywords "integrons", "antibiotic resistance", and *"Salmonella* Typhimurium". We included 38 articles reporting multidrug resistance up to five antibiotic families.

Class 1 integrons with *aadA2* and bla_PSE-1_ gene cassettes were predominant, some probably related to the *Salmonella* genomic island 1. We did not find studies detailing class 1 and 2 integrons in the same isolate, nor class 3 integrons reported. The presence of integrons largely explains the resistance profiles found in isolates from different sources in 15 countries.

A nivel mundial, *Salmonella* no tifoidea es el agente responsable de 157 millones de casos de gastroenteritis y 57.000 muertes, aproximadamente, y se considera un agente patógeno zoonótico importante, causante de enfermedades diarreicas y de enfermedades transmitidas por alimentos. La salmonelosis se manifiesta con síntomas clínicos como fiebre, dolor abdominal, diarrea, náuseas y, en algunos casos, enfermedad invasiva ^1^. *Salmonella* spp. se considera el agente patógeno de riesgo biológico más común en eventos de seguridad alimentaria; tan sólo en el año 2019, se reportó una frecuencia del 34 % ^2^.

*Salmonella* se divide en dos especies, *Salmonella bongori*y *Salmonella enterica. Salmonella enterica* se subdivide en siete subespecies, una de ellas es *enterica (S. enterica* subsp. *enterica)* y agrupa a la mayoría de las serovariedades que causan enfermedades en los humanos ^3^. A nivel global, las serovariedades de *Salmonella* más frecuentemente aisladas son Enteritidis y Typhimurium. Sin embargo, en la última década ha surgido un nuevo serotipo, la variante monofásica de Typhimurium (1,4,[5],12:i:-), que se ha convertido en una de las principales causas de salmonelosis transmitida de animales a humanos. Esta variante, a diferencia de *Salmonella* Typhimurium clásica, se caracteriza por la ausencia de la segunda fase flagelar. La infección por este nuevo serotipo se asocia generalmente con gastroenteritis, una enfermedad de resolución espontánea que con frecuencia no requiere el uso de antibióticos ^4^.

Typhimurium se subdivide en patovares (variantes patogénicas), algunos de los cuales presentan un amplio rango de huéspedes, como humanos, aves y animales de granja, y también se pueden recuperar de muestras ambientales como suelo y agua, mientras otras se restringen a ciertos huéspedes específicos. Convencionalmente, estos patovares se han identificado como fagotipos *(definitive fagotypes,* DT), pero en la era genómica se denominan tipos de secuencia *(sequence types,* ST) ^5^. El fagotipo DT104 es el más estudiado por su rápida diseminación a nivel global en animales de granja y en humanos; se caracteriza por ser resistente a la ampicilina, el cloranfenicol, la estreptomicina, las sulfonamidas y las tetraciclinas (perfil de resistencia conocido como ACSSuT, reportado a principios de la década de 1980). Además, el fagotipo DT104 tiene la capacidad de adquirir otros factores determinantes de resistencia de importancia clínica ^6^. Recientemente, se describió la aparición de la sublínea ST313 -también multirresistente, tipo ACSSuT- en África subsahariana, muy invasiva y cuya infección provoca cuadros clínicos de septicemia y meningitis ^7^.

El incremento de la resistencia a los antibióticos en Typhimurium es un problema importante de salud pública mundial. Esto se ha evidenciado en aislamientos de Typhimurium de origen humano, animal (pollos, bovinos, porcinos y pavos) y de carne para consumo, recolectados en Estados Unidos durante dos décadas (1996-2016), en los que predominan la resistencia a ampicilina, cloranfenicol, estreptomicina, sulfonamidas, tetraciclina, amoxicilina-ácido clavulánico, ceftriaxona, ceftiofur y, en menor proporción, a fluoroquinolonas ^8^. Sin embargo, el incremento de la resistencia a fluoroquinolonas y colistina convierten a esta serovariedad en un agente patógeno difícil de tratar en infecciones en humanos.

La resistencia reportada del serovar Typhimurium se encuentra codificada principalmente en integrones y transposones. Los integrones (figura 1, a y c) son plataformas de captura y expresión de genes, divididas en tres clases: clase 1 (int1), clase 2 (int2) y clase 3 (int3).

**Figura 1:**
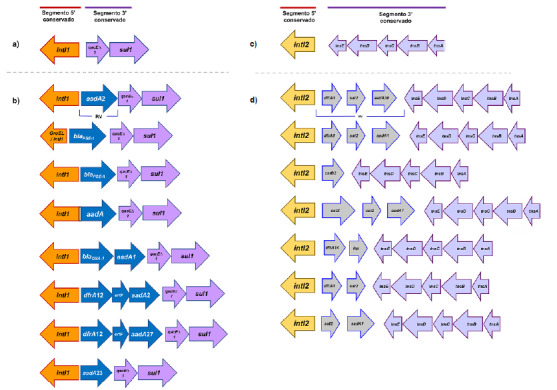
Esquema de los principales integrones de clase 1 (Int1) y de clase 2 (Int2) descritos en Typhimurium multirresistente. Las flechas de color naranja, purpura y azul-gris representan los extremos conservados 5' y 3' y la región variable de los Int1 e Int2, respectivamente. En a) y c), los extremos 5' y 3' conservados en los Int1 e Int2 clásicos; en b) y d), los principales Int1 e Int2 reportados junto con el arreglo de genes que conforman la RV.

Los integrones de clase 1 tienen tres regiones características; la primera es la secuencia conservada 5' (5'CS) que contiene el gen de la integrasa *intI1,* el promotor *Pc* que dirige la transcripción de los genes insertados en la estructura del integrón y el sitio de recombinación *attI1* reconocido por la integrasa para insertar los genes; la segunda corresponde a la región variable en la que se insertan los genes y al sitio de recombinación *attC;* y la tercera es el extremo conservado 3' (3'CS) generalmente conformado por los genes *qacEΔ1* y su/1 que le confieren resistencia a compuestos de amonio cuaternario y sulfonamidas, respectivamente.

Los integrones de clase 2 están comúnmente asociados con el transposón Tn7 y sus variantes, en su extremo 5'CS se encuentra el gen *intI*2 (no funcional debido a un codón de parada interno), dos promotores activos y el sitio de recombinación attI2; y en el extremo 3'CS contiene cinco genes *tns (tnsA, tnsB, tnsC, tnsD* y tnsE) que participan en la movilidad del transposón y el integrón mediante una inserción preferencial en un sitio único del cromosoma bacteriano ^9^^,^^10^. Los transposones son segmentos de ADN que tienen la capacidad de saltar a diferentes partes del material genético bacteriano por acción del gen de la transposasa y tienen la capacidad de transportar genes de resistencia, incluyendo integrones completos. Estas dos plataformas hacen parte de los elementos genéticos móviles capaces de diseminar genes de resistencia entre géneros bacterianos ^10^.

Como ejemplo de lo anterior, está la resistencia ACSSuT del DT104 (serovar Typhimurium) codificada en la isla genómica de *Salmonella* 1 (SGI1), localizada en el cromosoma con los genes *aadA2* y *bla*_*PSE-1*_ insertados en el integrón In104 ^6^; o la multirresistencia del genotipo ST313 codificada por un transposón de tipo Tn2í, insertado en el plásmido de virulencia de *Salmonella* (pSLT) ^11^.

Se han documentado aislamientos de Typhimurium ST19 multirresistente con integrones de clase 1, compuestos por genes de resistencia a los antibióticos, que pueden estar insertados en plásmidos o cromosomas. La presencia de integrones de clase 1 y de otros elementos genéticos móviles se ha considerado esencial para la transferencia horizontal de los factores determinantes de resistencia ^12^.

En Colombia, con el transcurso del tiempo, se ha reportado un aumento de la resistencia a antibióticos -como ampicilina, cefotaxima, ceftazidima, tetraciclina y, recientemente, ciprofloxacina- en aislamientos de Typhimurium recuperados de muestras clínicas ^13^^,^^14^. Sin embargo, la relación de dicha resistencia con la presencia de integrones ha sido poco explorada.

A nivel local, el conocer los factores genéticos determinantes implicados en la resistencia a los antibióticos en Typhimurium, aportará información para el plan nacional de respuesta a la resistencia a los antimicrobianos ^15^. Por lo tanto, el objetivo de esta revisión bibliográfica fue identificar los tipos de integrones y sus genes de resistencia en aislamientos multirresistentes de Typhimurium reportados durante 2012-2020 a nivel mundial.

## Desarrollo de la revisión y selección de artículos

Se realizó una revisión sistemática exploratoria en cinco etapas, según Arksey y O'Malley ^16^, en la cual se establecieron:

1. la pregunta de investigación bajo la estrategia PICO (población, intervención, control y resultados),
2. la identificación de estudios pertinentes;
3. la selección e inclusión de documentos;
4. la organización de los datos, y
5. la síntesis y el análisis de los resultados ^16^^,^^17^.

La búsqueda estuvo orientada por la pregunta: ¿cuáles son los tipos de integrones descritos en aislamientos multirresistentes a antibióticos de *Salmonella* Typhimurium?

Para la búsqueda efectiva, se utilizaron los operadores booleanos: AND, NOT, OR y XOR, y los descriptores en ciencias de la salud (Decs/ MeSH): *integrons, antibiotic resistance* y *Salmonella* Typhimurium, en todas las combinaciones posibles con las palabras clave en español o inglés. Se consultaron diferentes fuentes, como Medline, PubMed, SciELO, ScienceDirect, Redalyc y Google Académico, y se analizaron los artículos de revistas científicas publicados entre los años 2012 y 2020. La búsqueda se llevó a cabo entre noviembre y diciembre del 2020.

Los criterios de inclusión para seleccionar y clasificar los documentos como "pertinentes" fueron:

1. divulgación en el intervalo de tiempo examinado (2012-2020),
2. texto completo de la investigación original disponible en inglés o español,
3. registro del país y fuente de los aislamientos, y
4. descripción detallada del arreglo de genes en la región variable de los integrones junto con el perfil de resistencia de los aislamientos.

Para la selección de los artículos, solo se tuvieron en cuenta la sensibilidad y el contenido de integrones de los aislamientos bacterianos. No se consideraron los métodos de identificación del integrón ni el de sus genes, ni el proceso para determinar la sensibilidad ante los antibióticos. Aunque estos métodos pueden variar según el estudio, no afectan el objetivo principal de esta revisión.

Se excluyeron los artículos que estaban en un idioma diferente a español o inglés, los informes de casos clínicos, las opiniones de expertos, los comentarios de literatura, los artículos de revisión, los trabajos de grado y aquellos sin disponibilidad completa de la información descrita en los criterios de inclusión 3 y 4.

El perfil de resistencia a los antibióticos se estableció conforme a lo descrito por el programa *National Antimicrobial Resistance Monitoring System for Enteric Bacteria* (NARMS) de los *Centers for Disease Control and Prevention* (CDC) de los Estados Unidos, que lo definen como "una descripción de los resultados de las pruebas de susceptibilidad a los antibióticos para un aislamiento" ^18^. Los aislamientos reportados con resistencias a tres o más familias de antibióticos se definieron como multirresistentes ^19^.

Los resultados de las búsquedas fueron exportados y transferidos al administrador bibliográfico Sciwheel (https://sciwheel.com), instrumento que permite la organización de referencias. El proceso de lectura y evaluación de los documentos incluidos fue llevado a cabo por dos de los autores, con el fin de minimizar los riesgos de sesgo en la selección. En caso de desacuerdo en los hallazgos, se discutió el consenso entre los pares o se utilizó la apreciación de un tercer revisor.

Para la extracción, organización y análisis de la información obtenida, se aplicó un instrumento elaborado por los autores para caracterizar la información de las publicaciones con el uso de una matriz metodológica de síntesis, cuyas variables fueron: país de origen del aislamiento, fuente del aislamiento, resistencia por familia de antibióticos, arreglo de genes en la región variable de los integrones descritos y período de recolección de los aislamientos. Los datos recopilados en esta matriz se muestran en el cuadro suplementario 1. A continuación, se hizo un análisis del contenido para identificar los tipos de integrones asociados con las resistencias reportadas y la fuente.

**Figura 2:**
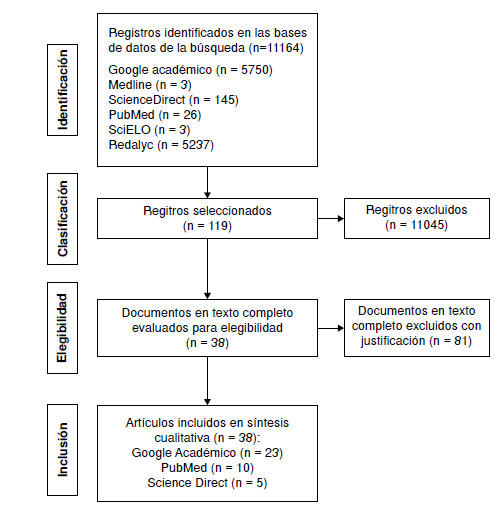
Diagrama de flujo del proceso de selección de artículos de la revisión

En la figura 2 se presenta el diagrama de flujo de acuerdo con el modelo PRISMA (20), que muestra el proceso de búsqueda y selección de los artículos que cumplieron con los criterios de inclusión.

## Características de los estudios

En la búsqueda inicial, resultaron 11.164 artículos provenientes de las seis bases de datos. En la fase de tamizaje, se eligieron 119 artículos de investigación que aparentemente cumplían con los cuatro criterios de inclusión enlistados en la sección de materiales y métodos. El texto completo de estos artículos fue revisado y analizado para confirmar que incluyeran la descripción de las características fenotípicas de resistencia para los aislamientos y las genotípicas para los integrones. Finalmente, solo 38 artículos presentaron una descripción clara de los datos y fueron incluidos en la matriz metodológica de síntesis para ser analizados.

Los artículos de investigación seleccionados tienen una amplia representación geográfica que incluye cuatro continentes, 15 países (Brasil, China, Corea del Sur, Egipto, España, Estados Unidos de América, India, Irán, Italia, Marruecos, Portugal, Reino Unido, República Checa, Tailandia, Taiwán) y la Unión Europea (cuadro suplementario 1).

En los 38 estudios publicados entre el 2012 y el 2020, se recopiló la información de 1.337 aislamientos de Typhimurium, recuperados entre los años 2002 y 2018; en cada estudio se analizaron de 1 a 191 aislamientos. El 34,2 % (n = 13) de los estudios reunía los aislamientos de los seis países asiáticos; el 31,6 % (n = 12) de los reportes contenía información de uno o más países europeos; el 26,3 % (n = 10), de los dos países africanos, y el 7,9 % (n = 3), de dos países del continente americano.

Según los criterios de interpretación del *Clinical and Laboratory Standards Institute,* la principal prueba de sensibilidad a antibióticos realizada en los trabajos seleccionados, fue el método de difusión en disco (73,7 %; n = 28), seguido por el método de microdilución (15,8 %; n = 6); en menor medida, se utilizó una combinación de los métodos de difusión en disco y microdilución (7,9 %; n = 3) y, finalmente, en un artículo (2,6 %) no se informó el método empleado.

Entre los 1.337 aislamientos estudiados, el 63,6 % (n = 850 / 1.337) correspondió a aislamientos multirresistentes, de los cuales cerca de la mitad portaban integrones de clase 1 (49,5 %; n = 421 / 850), todos ellos resistentes a antibióticos que abarcan entre tres a ocho familias diferentes; con menor frecuencia, se reportaron aislamientos con integrones de clase 2 (1,6 %; n = 14 / 850), resistentes a antibióticos pertenecientes a un rango de cuatro a ocho familias. En el 48,8 % (n = 415/850) de los aislamientos multirresistentes estudiados, no se encontraron integrones de clase 3.

De acuerdo con el objetivo del presente trabajo, a continuación, solo se describen los aislamientos multirresistentes de Typhimurium portadores de integrones.

## Multirresistencia en Typhimurium

Los 42 perfiles de resistencia encontrados entre los 435 aislamientos multirresistentes de Typhimurium, portadores de integrones, fueron nombrados con números romanos consecutivos (I-XLII), conforme se fueron documentando en los artículos.

Las familias de antibióticos más frecuentes fueron β-lactámicos (81 %; n = 34 / 42), aminoglucósidos e inhibidores de la vía del folato, ambos presentes en el 78,6 % (n = 33 / 42), tetraciclinas (66,7 %; n = 28 / 42) y anfenicoles (64,3 %; n = 27 / 42).

Globalmente, entre los aislamientos portadores de integrones, los más comunes fueron los resistentes a cinco familias de antibióticos (n = 260), seguidos por los resistentes a seis (n = 90), tres (n = 39), cuatro (n = 37) y siete (n = 8) familias de antibióticos. Solo se identificó un aislamiento resistente a ocho familias de antibióticos.

No fue posible identificar con exactitud el año con el mayor número de aislamientos multirresistentes portadores de integrones, pues en varios casos los autores solo reportaron un rango de tiempo que reúne todos los aislamientos analizados. Además, en 200 aislamientos de Typhimurium con multirresistencia a los antibióticos, no se documentó la fecha de obtención (cuadro suplementario 1).

**Figura 3:**
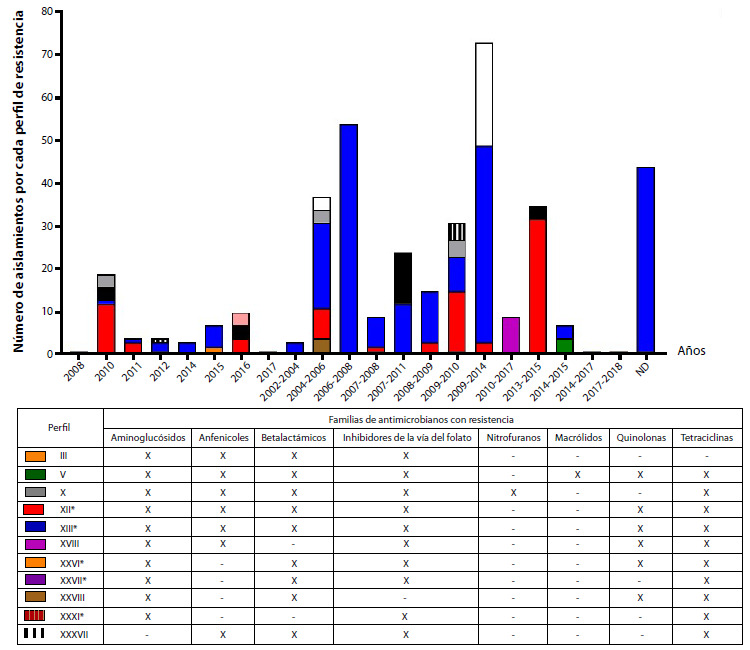
Distribución de aislamientos de Typhimurium multirresistente con integrones entre los principales perfiles de resistencia. La revisión de literatura mostró 42 perfiles diferentes de resistencia en aislamientos de Typhimurium. Se designaron con la nomenclatura en números romanos del I al XLII. En la gráfica se muestra la distribución de los 11 principales perfiles de resistencia en los diferentes años de estudio encontrados en los manuscritos revisados. Se observan dos grupos de datos, el primero conformado por un solo año y el segundo conformado por intervalos de años, este dato corresponde a las fechas de recuperación de los aislamientos reportados por los manuscritos incluidos en este estudio. En la tabla se muestra el respectivo perfil con su código de colores, y en las columnas las familias a las cuales presentan resistencia, en donde "X" es igual a resistente y "-" es igual a sensible. Los perfiles marcados con asteriscos corresponden a los cinco predominantes incluyendo el perfil XII (rojo) y XIII (azul).

En el periodo estudiado, los cinco perfiles de resistencia más frecuentes fueron el XII (n = 81), XIII (n = 222), XXVI (n = 21), XXVII (n = 10) y XXXI (n = 27), todos con resistencia a aminoglucósidos, inhibidores de la vía del folato y tetraciclinas. De estos, los perfiles XII y XIII, conformados por 303 aislamientos portadores de integrones, exhibieron resistencia común a aminoglucósidos, anfenicoles, p-lactámicos, inhibidores de la vía del folato y tetraciclinas. El perfil XII también fue resistente a quinolonas. Los perfiles XXVI, XXVII y XXXI reunían 58 aislamientos con resistencia a aminoglucósidos, β-lactámicos, inhibidores de la vía del folato y tetraciclinas, excepto el perfil XXXI que no mostró resistencia a los β-lactámicos. Se observó que el perfil de resistencia XIII prevaleció durante los 16 años de recolección, seguido del perfil XII que persistió en un rango de 11 años; los otros tres perfiles fueron intermitentes durante los años de análisis (figura 3).

**Figura 4:**
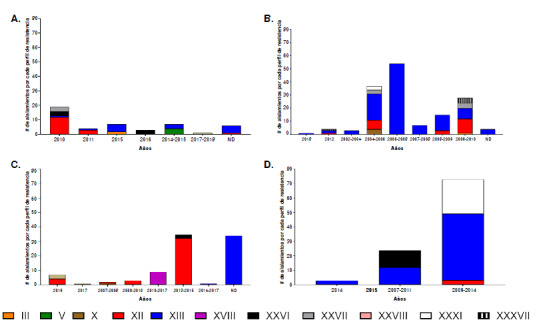
Principales perfiles de resistencia por familias de antimicrobianos en aislamientos de Typhimurium multirresistentes portadores de integrones publicados entre el 2012 y el 2020, por continente. En la gráfica se muestra el número de aislamientos encontrados por cada perfil de resistencia. Para este resultado solo se tuvieron en cuenta los principales perfiles por continente: (A) siete perfiles de África, (B) siete perfiles de Europa, (C) seis perfiles en Asia y (D) cuatro perfiles en América.

Los aislamientos de Typhimurium portadores de integrones se recuperaron de diversas fuentes, dependiendo del continente. En África, los aislamientos se recuperaron de animales de consumo, alimentos y humanos; fueron multirresistentes contra tres a ocho familias de antibióticos y presentaron 22 perfiles de resistencia, con preponderancia del XII y XIII, seguidos del XXVI, V, XXVII y III. El perfil III fue resistente a aminoglucósidos, anfenicoles, p-lactámicos e inhibidores de la vía del folato, mientras que el perfil V fue resistente a todas las familias incluidas en este estudio, excepto la de nitrofuranos (figuras 4a y 5).

**Figura 5:**
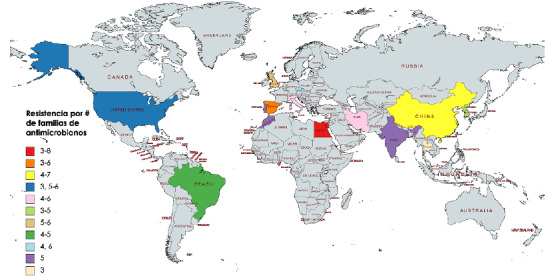
Número de resistencias por familias de antibióticos en países de cuatro continentes publicados entre el 2012 y el 2020 (elaborado con MapChart https://mapchart.net/world.html). El color de cada país indica el número de las resistencias por familias de antibióticos en los aislamientos de Typhimurium multirresistente portadores de integrones.

En Europa, las cepas fueron aisladas de animales de consumo, aves silvestres, plantas de tratamiento de aguas residuales y humanos; estas fueron multirresistentes a los antibióticos de tres a siete familias y revelaron 15 perfiles de resistencia: los dos principales fueron XII y XIII, seguidos del XXVII, XXXVII y X. El perfil X es similar al perfil XII al reemplazar quinolonas por nitrofuranos. Los datos reportados para este continente proceden de la Unión Europea y de otros cinco países de la región (figuras 4b y 5).

En Asia, los aislamientos se obtuvieron de animales de consumo, animales silvestres, alimentos y humanos, y provenían en su mayoría de China, seguida de Corea del Sur, Irán y otros tres países de la región; fueron multirresistentes a antibióticos de tres a siete familias y evidenciaron 17 perfiles de resistencia, entre los que sobresalieron el XII y el XIII, seguidos del XVIII, el XXXVIII y el XXVI (figuras 4c y 5).

En América, las cepas fueron aisladas de animales de consumo y de humanos. Los aislamientos fueron multirresistentes a antibióticos de tres a seis familias diferentes y presentaron cinco perfiles de resistencia, con el perfil XIII como predominante, seguido del XXXI, el XXVI y el XII. Los datos registrados provienen de Brasil y Estados Unidos entre 2016 y 2020 (figuras 4d y 5).

El surgimiento y la diseminación de cepas de Typhimurium multirresistentes a antibióticos de tres o más familias, provenientes de países de cuatro continentes, se evidenció durante los 16 años de recolección que abarcó esta revisión.

## Identificación de integrones presentes en cepas de Typhimurium multirresistentes a antibióticos

En los 38 artículos seleccionados, publicados entre el 2012 y el 2020, se reportaron 421 aislamientos portadores de 615 integrones de clase 1, con 38 arreglos de genes diferentes en su región variable; y 14 aislamientos con 17 integrones de clase 2 que tienen 7 arreglos de genes en su región variable. En ninguno de los 435 aislamientos multirresistentes de Typhimurium se encontraron integrones de clase 3, ni presencia simultánea de integrones de clase 1 y 2 (cuadro suplementario 1).

Entre los integrones de clase 1, los genes más frecuentes en la región variable fueron bla_PSE-1_ (n = 162), *aadA2* (n = 142) y *aadA* (n = 68) como casetes independientes y, los arreglos *bla*_OXA-1_ *-aadA1* (n = 55) y *dfrA12-orfF-aadA2* (n = 35), como grupos de genes en casetes. Se observó que la mayoría de las cepas resistentes de Typhimurium contenían integrones de clase 1 (96,8 %; n = 421/435), conformados por regiones variables dispuestas en arreglos -de uno a cuatro casetes de genes- en un mismo aislamiento. En el cuadro 1 se muestran los genes encontrados en los cinco perfiles de resistencia más sobresalientes de los integrones de clase 1.

Se identificaron 117 aislamientos que portaban dos integrones independientes de clase 1: uno con el gen de resistencia *aadA2* y otro con el bla_PSE-1_. Las metodologías empleadas para la identificación de genes y el fenotipo de pentarresistencia ACSSuT sugieren que, de estas cepas, 70 probablemente estén asociadas con la isla genómica SGI1 (de localización cromosómica) y sean representantes del clon multirresistente DT104 de Typhimurium. La mayor parte de estas cepas exhibieron los perfiles de resistencia más abundantes (XIII, n = 48; y XII, n = 20). Un análisis posterior de estos aislamientos por WGS *(Whole Genome Sequencing)* confirmó la presencia de la SGI1 y su constante circulación a nivel global.

El análisis de la relación de los integrones de clase 1 con los perfiles de resistencia, mostró que, en el perfil XIII, los genes imperantes fueron b/a_PSE-1_ (n = 140), *aadA2* (n = 100), *aadA* (n = 44) y bla_*OXA-1*_ *-aadA1* (n = 36); para el perfil XII, fueron *dfrA12-orfF-aadA2* (n = 27), *aadA2* (n = 25) y *bla*_PSE-1_ (n = 19); en el perfil XXXI, el gen más frecuente fue de la familia *aadA;* y en el perfil XXVI, el arreglo imperante fue *dfrA12-orfF-aadA27* (n = 12), mientras que, en el perfil XXVII, se destacaron los genes de la familia *dfrAI* (n = 4) (cuadro 1) (figura 1b).

**Cuadro 1:** Integrones de clase 1 descritos en aislamientos de Typhimurium multirresistente en los cinco perfiles de resistencia predominantes

RV-Int1 (5'-3')	Perfil de resistencia				Total Int1
	XII	XIII	XXVI	XXVII XXXI	
*aac(6')-Ib-cr-blaOXA-1-catB3-arr3*		2			2
*aadA*		44		24	68
*aadAI*	1			1	2
*aadA1-like-aadA2-dfrA12*	1	6		1	8
*aadA2*	25	100	1	1	127
*aadA23*		12			12
*aadA2-blaPSE-1*	1	2			3
*aadA6-orfD*	3				3
*aadA7-aac(3)-Id*			3		3
*aadA-dfrA12*	3	2			5
*aadB-catB3*		8			8
*blaOXA-1-aadA1*	9	36			45
*blaPSE-1*	19	140			159
*dfrA1*				4	4
*dfrA12-aadA2*	1				1
*dfrA12-aadA2-cmlA1-aadA1*	2	5			7
*dfrA12-orfF-aadA2*	27	3	4	1	35
*dfrA12-orfF-aadA27*		2	12		14
*dfrA12-orfF-aadA2-cmlA1-aadA1*		1			1
*dfrA15*		4		1	5
*dfrA15b-cm1A4-aadA2*	1				1
*dfrA17-aadA5*	4				4
*dfrA1-aadA1*	1	5	1	1	8
*drf12-orfF-aadA2*		3			3
*estX-psp-aadA2-cmlA1-aadA1*		1			1

Int1: integrón de clase 1; RV: región variable

Entre los integrones de clase 2, se encontraron siete arreglos de genes y *dfrA1-sat2-aadA30* fue el más frecuente (cuadro 2) (figura 1d).

Los resultados obtenidos sugieren que las resistencias descritas de tres a ocho familias de antibióticos en los perfiles predominantes podrían estar codificadas por los genes contenidos en los integrones de tipo 1 y 2 (cuadros 1 y 2).

**Cuadro 2:** Integrones de clase 2 descritos en aislamientos de Typhimurium multirresistente

RV-Int2 (5'-3')	Perfil de resistencia										Total Int2
	IV	VI	VII	XI	XII	XIII	XXIII	XXVII	XXXIV	XXXVI	
*catB2*									1	1	2
*dfrA14- lsp*				1							1
*dfrA1-sat2*								1			1
*dfrA1-sat2-aadA1*					2	1					3
*dfrA1-sat2-aadA30*	1	1	2				1		1	1	7
*estX-sat2-aadA1*								2			2
*sat2-aadA1*									1		1

Int2: integrón de clase 2; RV: región variable

En la tabla se muestran los perfiles de resistencia y la región variable de los integrones de clase 2 (Int2). El número total de Int2 es superior al número de aislamientos reportados debido a que dos aislamientos portan más de 1 integrón.

Esto concuerda con los estudios que reportan a los genes de tipo *aadAs* como responsables de generar resistencia específica a estreptomicina (de la familia de los aminoglucósidos), pues los valores de concentración inhibitoria mínima se incrementan de 16 a 128 veces. Asimismo, el gen bla_OXA-1_ confiere resistencia a amoxicilina (β-lactámicos), los genes *dfrA* proporcionan altos niveles de resistencia a trimetoprima (inhibidor de la vía del folato) y los genes *catB* conceden resistencia a cloranfenicol (anfenicoles).

En la tabla se muestran los cinco perfiles de resistencia predominantes y la región variable de los integrones de clase 1 (Int1). El número total de Int1 es superior al número de aislamientos reportados debido a que algunos aislamientos portan entre 2 y 4 integrones.

La relación de la expresión de estas resistencias con los genes mencionados, así como con otros de las mismas familias, ha sido reportada por otros autores ^21^^,^^22^. La resistencia a tetraciclinas, nitrofuranos y macrólidos, por lo general, no está asociada con integrones, sino con transposones o plásmidos conjugativos; por esta razón, no se discute su relación con los perfiles de resistencia ^23^^,^^24^.

En esta revisión se observó que los genes presentes en los integrones más frecuentes pertenecían principalmente a las familias *aadAy dfrA,* lo cual concuerda con la mayoría de los estudios sobre integrones de Enterobacteriaceae de hace varias décadas ^25^^,^^26^. Entre los 615 integrones descritos, solo se encontraron 38 arreglos diferentes, lo cual concuerda con estudios genómicos recientes que demuestran una diversidad relativamente escasa de integrones en *Salmonella*^27^.

El perfil de resistencia no siempre está dado por el mismo integrón; por esto, los perfiles XII y XIII tienen un mayor número de arreglos de integrones de clase 1 y comparten los genes predominantes, mientras que los perfiles XXVI, XXVII y XXXI presentan genes y arreglos diferentes.

En cuanto a los integrones de clase 2, se observó que los perfiles preponderantes XII, XIII y XXVII se deben también a arreglos genéticos, pero presentes en menor proporción que en los integrones de clase 1. Cabe mencionar que no se observó ninguna relación destacable entre los arreglos y los perfiles de resistencia. Similar a lo observado en los integrones de clase 1, la mayoría de los genes fueron de las familias *dfrA, sat* y *aadA.* La mayoría de los integrones de clase 2 se describieron en aislamientos africanos (n = 12), distribuidos en nueve perfiles de resistencia, entre los que se sobresalen los perfiles XXXIV (n = 3) y XXXVI (n = 2) por reunir mayor número de aislamientos portadores de estos integrones; en los perfiles restantes, por cada uno solo se reportó un aislamiento con integrones de clase 2. Solo dos aislamientos con integrones de clase 2 se reportaron en Asia (n = 1) y Europa (n = 1). Esto contrasta con lo observado en los integrones de clase 1, donde el mayor número de aislamientos se reportó en Europa (n = 163), seguida de Asia (n = 106), América (n = 101) y África (n = 65). Además, se resalta que en África es donde se describen más integrones de clase 1 (n = 19), seguida de Asia (n = 17), Europa (n = 14) y América (n = 6) (cuadro suplementario 1).

La serovariedad Typhimurium es uno de los principales agentes causantes de salmonelosis en humanos y animales a nivel global ^28^^,^^29^. Actualmente, las cepas multirresistentes de Typhimurium se consideran un problema de salud pública, ya que su transmisión mediante la cadena alimentaria ocasiona brotes de intoxicación de difícil tratamiento. Por esta razón, se considera una problemática que debe abordarse desde el enfoque de Una salud (One *health)*^30^^,^^31^. Desde que, en 1968, Anderson ^32^ registró la diseminación del clon multirresistente de Typhimurium DT29 en el Reino Unido hasta la posterior diseminación global de DT104, se ha evidenciado que la presencia de integrones de clase 1 es clave en la propagación exitosa de estos clones ^6^^,^^32^^,^^33^. Actualmente, son los elementos genéticos móviles predominantes de esta serovariedad ^27^.

Desde entonces, numerosos reportes han evidenciado la presencia de integrones en *Salmonella* spp., insertados en el cromosoma o presentes en plásmidos, recuperados de diversas fuentes a lo largo de la cadena alimentaria y en diferentes países, lo que resalta la importancia de estudiar y documentar estos mecanismos.

En el presente trabajo, el análisis de los datos obtenidos de 38 artículos, provenientes de cuatro continentes, 15 países y la Unión Europea, permitió caracterizar la resistencia a antibióticos asociada con integrones en cepas multirresistentes de Typhimurium y su desarrollo a lo largo de nueve años de divulgación científica.

Los resultados de esta revisión muestran principalmente la presencia de integrones de clase 1 y, en menor medida, de integrones de clase 2, portadores de diversos genes de resistencia en el 52 % (n = 449 / 864) de los aislamientos multirresistentes de Typhimurium, recuperados de diversas fuentes, como animales de consumo y silvestres, alimentos, muestras clínicas de humanos y aguas residuales de plantas de tratamiento, lo que muestra su amplia dispersión (cuadro suplementario 1).

Los datos recopilados en esta revisión muestran que, tanto la multirresistencia a los antibióticos como la presencia de integrones, se han incrementado mundialmente en Typhimurium con el paso de los años. Así, entre el 2002 y el 2004, las cepas aisladas solo describen dos perfiles de resistencia, mientras que las recolectadas en años posteriores muestran más: 11 perfiles en los aislamientos del 2004 al 2006, cinco en los del 2007 al 2011, 14 perfiles identificados entre el 2015 y el 2016, y nueve en las cepas del 2016 y el 2017. Durante los periodos de recolección, se evidenció resistencia constante a aminoglucósidos, p-lactámicos, inhibidores de la vía del folato y tetraciclinas (cuadro suplementario 1).

Aunque no está vinculada con integrones, es importante el registro de resistencia a quinolonas que reportan algunos autores en varios de los perfiles predominantes en África, Asia y las Américas, lo que concuerda con la alerta por la emergencia y la diseminación de estos genes en el género *Salmonella*^34^^,^^35^.

Estas observaciones sugieren dos posibles escenarios: que existe una circulación global de aislamientos con determinados genes de resistencia o que hay un recambio de genes de resistencia en los aislamientos de cada región geográfica. La secuenciación del genoma completo en estas cepas sería una excelente herramienta para estudiar el trasfondo genético en el que interactúan los factores determinantes de resistencia y para analizar posibles relaciones filogenéticas.

El surgimiento y la diseminación de aislamientos de Typhimurium multirresistentes a antibióticos en países de diferentes continentes, se han evidenciado por más de 30 años ^36^^-^^38^. Tal es el caso de las cepas relacionadas con los genes predominantes *bla*_PSE-1_ *, aadA2, aadA, bla*_OXA-1_ *-aadA1* y *dfrA12-orfF-aadA2,* o con los menos frecuentes, como *cmlAI* y *catB*4, que confieren resistencia a un grupo limitado de familias de antibióticos, como β-lactámicos, aminoglucósidos, inhibidores de la vía del folato y anfenicoles. En el 2009, Partridge *et al.* revisaron 130 casetes de integrones reportados en el GenBank del NCBI *(National Center for Biotechnology Information),* y resaltaron a los genes *aacA4* y *aadB* por su papel en la resistencia a aminoglucósidos *(aacA4* a gentamicina y amikacina; *aadB* a gentamicina) ^39^. Estos datos indican que los casetes de genes descritos pueden estar compartidos en el acervo genético de varias especies de enterobacterias, incluida Typhimurium.

Los resultados de esta revisión sugieren que los países africanos y europeos tienen más interés en este tipo de estudios, al encontrar un mayor número de publicaciones provenientes de estos continentes, mientras que en América los datos son limitados. Al respecto, solo un estudio de Brasil y dos de Estados Unidos, relacionados con aislamientos de Typhimurium y su multirresistencia asociada con integrones, cumplieron con los criterios de inclusión descritos en los métodos.

En Colombia, existen reportes de cepas de *Salmonella* spp. Resistentes, al menos, a un antibiótico; estas fueron aisladas a partir de heces de porcinos previamente recolectadas de camiones de transporte y corrales. ^40^. También, se ha hecho seguimiento a aislamientos humanos de *Salmonella* spp. de origen clínico, mediante vigilancia -por pruebas de laboratorio-desarrollada en forma pasiva y voluntaria por entidades prestadoras de servicios de salud, como los laboratorios de salud pública departamental y el Instituto Nacional de Salud.

En estos esfuerzos aislados, se ha descrito que el serovar Typhimurium presenta los porcentajes más altos de resistencia a β-lactámicos, anfenicoles, tetraciclinas e inhibidores de la vía del folato, con una tendencia en aumento a lo largo del tiempo ^14^. Sin embargo, no se identificaron estudios entre el 2012 y el 2020 con la descripción de integrones, lo que sugiere que su estudio en Colombia y en otros países de la región no fue considerado de relevancia para el conocimiento, manejo y control de la resistencia en este serovar.

No obstante, en un estudio del 2006 en Colombia, se encontraron cepas multirresistentes de Typhimurium con integrones de clase 1 en el 39 % (n = 153 / 392) de los aislamientos clínicos y el 22 % (n = 11 / 50) de los porcinos. Se identificaron 12 casetes con genes diferentes, con el arreglo dfr7-aac(secuencia parcial)-bla_OXA-2_ como el más frecuente. Este integrón había sido previamente reportado en Colombia ^41^ como portador de genes que confieren resistencia a inhibidores de la vía del folato, aminoglucósidos y β -lactámicos.

En el estudio de Flórez-Delgado *et al.,* se reportó la presencia de este integrón en el 75,2 % (n = 115 / 153) de los aislamientos clínicos con integrones de clase 1 y que ha circulado en el país por 20 años (1997-2017) ^42^. La comparación de estos resultados con los obtenidos de la revisión de la literatura reciente destaca dos características distintivas de los integrones encontrados en Colombia:

- los genes *dfr7* y bla_OXA-2_ no son comunes en otros aislamientos del serovar Typhimurium, y
- el gen bla_OXA-2_ se ubica en el extremo 3', mientras que la posición más común para el gen bla_OXA-1_ es en el extremo 5' o en la mitad de la región variable.

La posición del gen en el casete del integrón podría estar influenciada por el fenotipo de resistencia que confiere y la presión selectiva ejercida por las actividades humanas, como el uso de antibióticos como promotores de crecimiento en animales de granja o para el tratamiento de casos clínicos ^39^.

Es interesante que el integrón predominante en aislamientos colombianos de Typhimurium desde hace 20 años esté compuesto por genes con una distribución poco común en esta serovariedad, lo que puede sugerir que se ha mantenido en la población de manera endémica. Estudios complementarios de integrones en otras especies bacterianas o de aislamientos más antiguos, podrían ayudar a descifrar el origen de este integrón.

Los hallazgos descritos muestran la importancia de conocer las características principales de los integrones que circulan en Colombia y su relación con la expresión de resistencia. Esta información podría considerarse en el diseño de acciones de control de la resistencia antibiótica en diferentes ambientes y fuentes.

La aparición de cepas resistentes y multirresistentes de *Salmonella* spp., tiene muchas consecuencias clínicas y en salud pública, asociadas con la falla terapéutica, lo que limita la elección del tratamiento y aumenta los brotes, la carga de la enfermedad y, posiblemente, la virulencia de las cepas, sin contar con el aumento de la mortalidad y la morbilidad, el alza en los costos del tratamiento, el incremento de la permanencia en los hospitales -y el riesgo de adquisición de infecciones intrahospitalarias-, así como el aumento de la transmisión de cepas multirresistentes de *Salmonella* spp. ^21^^,^^43^.

Por esta razón, se ha seleccionado a *Salmonella* spp. como uno de los agentes patógenos principales que se deben vigilar en el enfoque "Una salud" (One *health),* ya que su diseminación permite hacer seguimiento de la transmisión, en la cadena agroalimentaria hasta el humano, del microorganismo y de los factores determinantes de la resistencia.

Por todo lo anterior, en el mundo se han emitido normas que regulan el uso de los agentes antimicrobianos de importancia crítica en el área veterinaria, la producción de alimentos y la salud pública, prohibiendo usos diferentes a los registrados en la etiqueta del medicamento ^21^^,^^43^. También, se han establecido programas de vigilancia de la resistencia a los antibióticos, algunos de los cuales integran los resultados de aislamientos de origen humano, animal y alimentario, como el *National Antimicrobial Resistance Monitoring System for Enteric Bacteria (NARMS)* en los Estados Unidos de América y el *Danish Integrated Antimicrobial Resistance Monitoring and Research Programme (DANMAP)* en Dinamarca. Con programas en colaboración con los CDC, la Organización Mundial de la Salud (OMS) y la *Food and Drug Administration* (FDA) de los Estados Unidos, se ha rastreado la incidencia de la resistencia, especialmente de agentes patógenos relacionados con brotes de enfermedades transmitidas por alimentos en el mundo, para que las entidades competentes adopten estrategias, medidas de vigilancia y control, decisiones legales, sociales y financieras, en pro de combatir la amenaza que representa la resistencia a antibióticos en patógenos de interés en salud pública ^21^.

Debido a la importancia que los integrones de clases 1 y 2 juegan en la multirresistencia de *Salmonella* spp. a antibióticos, el conocimiento de estos mecanismos genéticos puede aportar información que apoye las acciones de vigilancia y control necesarias para evitar su diseminación.

## Conclusiones

La emergencia de la resistencia a los antibióticos en *Salmonella* spp. es multifactorial (condiciones externas y genéticas) y entre los elementos que influyen en su desarrollo se encuentra el uso de antibióticos que puedan ejercer una presión selectiva positiva hacia cepas bacterianas mejor adaptadas para sobrevivir a factores ambientales cambiantes. Esto, junto con la transferencia de genes de resistencia a serovariedades como Typhimurium, ha contribuido al surgimiento y propagación de la multirresistencia a los antibióticos y, por ende, al incremento de los costos en la salud pública y la práctica clínica por la ineficacia de los tratamientos disponibles.

En el presente trabajo, se han compilado los estudios que han identificado aislamientos multirresistentes de Typhimurium en diferentes países, recuperados de diferentes fuentes humanas, animales y ambientales, sus perfiles de resistencia a los antibióticos y sus integrones asociados. La resistencia a cinco familias de antibióticos -aminoglucósidos, anfenicoles, β-lactámicos, inhibidores de la vía del folato y tetraciclinas- se mantiene en el tiempo y tiene una circulación mundial.

Se confirmó que, en cepas multirresistentes de Typhimurium, aún no se reporta la presencia de integrones de clase 3; sin embargo, los integrones de clase 1 son los más frecuentes y no se reportan aislamientos que contengan a la vez integrones de clase 1 y 2. Los integrones encontrados explican en gran medida, mas no en su totalidad, los perfiles de resistencia de los aislamientos y se pudo evidenciar que diferentes integrones pueden proporcionar un mismo perfil de resistencia (cuadro suplementario 1).

### Recomendaciones

Al considerar que constantemente surgen cepas multirresistentes de Typhimurium que se diseminan en las regiones y el mundo, es necesario profundizar en el estudio de los elementos genéticos que aportan al incremento y movilización de los factores determinantes de resistencia en *Salmonella* y otras especies bacterianas de importancia en salud pública. Esta información puede ser utilizada en estrategias regionales y globales enfocadas en el control y en la disminución de la resistencia bacteriana en los ecosistemas silvestres, rurales, y urbanos, la producción animal y alimentaria, y en la salud humana. Estas acciones deben ser conjuntas, pues las iniciativas individuales no generan el impacto esperado a largo plazo para controlar este problema de interés en salud pública.

Una estrategia importante por aplicar es el control eficaz del manejo y uso de antibióticos de importancia crítica para la medicina humana -como los carbapenémicos, las quinolonas y otros ^44^- en la cría, la producción de alimentos y la salud animal. Se recomienda realizar pruebas piloto en las que se elimine el uso de algunos de estos antibióticos y se mida -a corto, mediano y largo plazo- el impacto de estas medidas sobre la resistencia a los agentes antibacterianos en el ambiente y los animales. Estas directrices podrían contribuir al control y la reducción de la multirresistencia y, por ende, favorecer la eficacia de los antibióticos disponibles para el tratamiento de bacterias resistentes que afecten la salud humana.

Aunque en la medicina humana existen medidas de control que regulan el uso de los antibióticos, es necesario reforzar las estrategias de educación para concientizar a la población de la importancia de no automedicarse, y de usar los antibióticos en la forma y dosis correctas para el tratamiento de las enfermedades. Otro aspecto por considerar es la revisión adecuada, eficaz y actualizada de la administración de antibióticos para el tratamiento de las enfermedades bacterianas en los hospitales, las clínicas ambulatorias, los centros médicos y la comunidad en general, para que esté en concordancia con los datos actualizados de la vigilancia de la resistencia a los antibióticos en la salud humana, animal y ambiental.

En esta revisión, se buscaron artículos publicados sobre la resistencia a antibióticos y su asociación con la presencia de integrones en aislamientos multirresistentes de Typhimurium y se filtraron, con base en cuatro criterios, para obtener información detallada y comparable entre ellos. Llama la atención que solo el 0,3 % de las publicaciones cumplieron con la inclusión de los datos del origen, la fecha, el perfil de resistencia a antibióticos, la presencia de integrones y la descripción de los casetes de genes.

Por todo lo anterior, se invita a los investigadores de esta área a hacer un esfuerzo para incluir la mayor cantidad de información sobre el origen de los aislamientos (metadatos), así como las características fenotípicas y genotípicas de las resistencias, para promover la compilación de mayor conocimiento de la resistencia a los antibióticos en este serovar.

### Limitaciones

En primer lugar, la fecha de publicación de los artículos incluidos en esta investigación es posterior a la obtención de los aislamientos. Esta discrepancia temporal podría afectar la interpretación de los resultados, ya que los hallazgos no reflejarían la situación del momento exacto de la recolección de las muestras.

Otra limitación relevante se relaciona con el reporte de las fechas de obtención de los aislamientos. En muchos casos, estas fechas se agrupan en bloques o no se presentan de manera individual con su respectivo registro. Esta falta de detalle dificulta un análisis exhaustivo de los integrones y su persistencia a lo largo del tiempo.

Aunque existen numerosos estudios sobre integrones en diferentes partes del mundo, no todos cumplen con los criterios de inclusión establecidos en esta revisión. Algunas publicaciones carecen de una descripción minuciosa de los integrones o no especifican el origen de los aislamientos. Esta omisión resulta en una pérdida de datos valiosos que podrían ser relevantes para el estudio de los integrones. Por lo tanto, se alienta a los investigadores a incluir una matriz de datos primarios más completa en sus futuros trabajos.

## Archivos suplementarios

**Cuadro suplementario 1:** Multirresistencia a los antimicrobianos e integrones en Typhimurium provenientes de aislamientos de origen humano, animal, de alimentos y ambiental publicados entre el 2012 y el 2020.

País	Origen	Resistencia por familias de antibiótico	Perfil de resistencia	Aislamientos multirresistente con Int (Total aislamientos multirresistente)	RV-Int1 (5'-3')	RV-Int2 (5'-3')	Años de recolección	Referencia
España	Humano	Aminoglucósidos, anfenicoles, betalactámicos, inhibidores de la vía del folato, tetraciclinas	XIII	3 (5)	*bla*_OXA-1_ *-aadA1*		2002-2004	(1)
España	Pollo, cerdo	Aminoglucósidos, anfenicoles, betalactámicos, inhibidores de la vía del folato, tetraciclinas	XIII	4 (4)	*bla*_OXA-1_ *-aadA1*		ND	(2)
Reino Unido	Cerdo	Aminoglucósidos, anfenicoles, betalactámicos, inhibidores de la vía del folato, tetraciclinas	XIII	5 (14)	*dfrA12-aadA2-* *cmlA1-aadA1*		2004-2006	(3)
Unión Europea	Cerdo	Aminoglucósidos, anfenicoles, betalactámicos, inhibidores de la vía del folato, tetraciclinas	XIII	8 (43)	*aadA2; hla*_PSE-1_		2004-2006	(4)
Unión Europea	Cerdo	Aminoglucósidos, anfenicoles, betalactámicos, inhibidores de la vía del folato, tetraciclinas	XIII	1 (43)	*bla*_OXA-1_ *-likeaadA1*		2004-2006	(4)
Unión Europea	Cerdo	Aminoglucósidos, anfenicoles, betalactámicos, inhibidores de la vía del folato, tetraciclinas	XIII	6 (43)	*aadA1-likeaadA2-* *dfrA12*		2004-2006	(4)
Italia	Humano	Aminoglucósidos, anfenicoles, betalactámicos, inhibidores de la vía del folato, tetraciclinas	XIII	46 (62)	*aadA2, hla*_PSE-1_		2006-2008	(5)
Italia	Humano	Aminoglucósidos, anfenicoles, betalactámicos, inhibidores de la vía del folato, tetraciclinas	XIII	6 (62)	*bla*_*OXA-1-*_ *aadA1*		2006-2008	(5)
Italia	Humano	Aminoglucósidos, anfenicoles, betalactámicos, inhibidores de la vía del folato, tetraciclinas	XIII	2 (62)	*aadA2; bla*_PSE-1_ *;* *dfrA1-aadA1*		2006-2008	(5)
Portugal	Cerdo	Aminoglucósidos, anfenicoles, betalactámicos, inhibidores de la vía del folato, tetraciclinas	XIII	7 (25) *	*aadA2; bla*_PSE-1_		2007-2008	(6)
Brasil	Cerdo	Aminoglucósidos, anfenicoles, betalactámicos, inhibidores de la vía del folato, tetraciclinas	XIII	12 (45)	*aadA23*		2007-2011	(7)
Italia	Humano	Aminoglucósidos, anfenicoles, betalactámicos, inhibidores de la vía del folato, tetraciclinas	XIII	1 (1)	*dfrA12-orfF-* *aadA2-cmlA1-* *aadA1*		2008	(8)
España	Cerdo	Aminoglucósidos, anfenicoles, betalactámicos, inhibidores de la vía del folato, tetraciclinas	XIII	2 (46)	*aadA2-hla*_PSE-1_		2008-2009	(9)
España	Cerdo	Aminoglucósidos, anfenicoles, betalactámicos, inhibidores de la vía del folato, tetraciclinas	XIII	10 (46)	*bla*_*OXA-1-*_ *aadA1*		2008-2009	(9)
España	Humano	Aminoglucósidos, anfenicoles, betalactámicos, inhibidores de la vía del folato, tetraciclinas	XIII	3 (55)	*bla*_*OXA-1-*_ *aadA1*		2009-2010	(10)
España	Humano	Aminoglucósidos, anfenicoles, betalactámicos, inhibidores de la vía del folato, tetraciclinas	XIII	2 (55)	*aadA2; hla*_*PSE-1*_		2009-2010	(10)
España	Humano	Aminoglucósidos, anfenicoles, betalactámicos, inhibidores de la vía del folato, tetraciclinas	XIII	2 (55)	*dfrA1-aadA1*		2009-2010	(10)
España	Humano	Aminoglucósidos, anfenicoles, betalactámicos, inhibidores de la vía del folato, tetraciclinas	XIII	1 (55)	*estX-psp-* *aadA2-cmlA1-* *aadA1*		2009-2010	(10)
Estados Unidos de América	Humano	Aminoglucósidos, anfenicoles, betalactámicos, inhibidores de la vía del folato, tetraciclinas	XIII	1 (101) t	*bla*_PSE-1_ *o* *bla*_CARB-6_		2009-2014	(11)
Estados Unidos de América	Humano, cerdo, bovino	Aminoglucósidos, anfenicoles, betalactámicos, inhibidores de la vía del folato, tetraciclinas	XIII	43 (101) †	*aadA1, aadA2* *o aadA3;* *bla*_PSE-1_ *o* *blaCARB-6*		2009-2014	(11)
Estados Unidos de América	Humano	Aminoglucósidos, anfenicoles, betalactámicos, inhibidores de la vía del folato, tetraciclinas	XIII	1 (101) †	*aadA1, aadA2* *o aadA3;* *blaPSE-1 o* *blaCARB-6;* *aadA1, aadA2* *o aadA3-* *dfrA12*		2009-2014	(11)
Estados Unidos de América	Bovino	Aminoglucósidos, anfenicoles, betalactámicos, inhibidores de la vía del folato, tetraciclinas	XIII	1 (101) †	*aadA1, aadA2* *o aadA3-* *dfrA12*		2009-2014	(11)
Egipto	Carne de res	Aminoglucósidos, anfenicoles, betalactámicos, inhibidores de la vía del folato, tetraciclinas	XIII	1 (24)	*dfrA1-aadA1*		2010	(12)
Egipto	Pollo	Aminoglucósidos, anfenicoles, betalactámicos, inhibidores de la vía del folato, tetraciclinas	XIII	1 (6)	*dfrA1-aadA1*		2011	(13)
Portugal	Humano	Aminoglucósidos, anfenicoles, betalactámicos, inhibidores de la vía del folato, tetraciclinas	XIII	2 (2)	*aac(6')-Ib-crbla*_OXA-__1_ *-* *catB3-arr3*		2012	(14)
Estados Unidos de América	Humano	Aminoglucósidos, anfenicoles, betalactámicos, inhibidores de la vía del folato, tetraciclinas	XIII	3 (3)	*aadA2b;*		2014	(15)
Egipto	Pollo	Aminoglucósidos, anfenicoles, betalactámicos, inhibidores de la vía del folato, tetraciclinas	XIII	3 (58)	*dfrA15; aadA2*		2014-2015	(16)
Egipto	Pollo	Aminoglucósidos, anfenicoles, betalactámicos, inhibidores de la vía del folato, tetraciclinas	XIII	2 (21)	*dfrA15; dfrA12-* *orfF-aadA27*		2015	(17)
Egipto	Pollo	Aminoglucósidos, anfenicoles, betalactámicos, inhibidores de la vía del folato, tetraciclinas	XIII	1 (21)	*dfrA15; aadA2;* *aadA1; dfrA12-* *orfF-aadA27*		2015	(17)
Egipto	Humano	Aminoglucósidos, anfenicoles, betalactámicos, inhibidores de la vía del folato, tetraciclinas	XIII	1 (21)	*aadA2*		2015	(17)
Egipto	Pollo	Aminoglucósidos, anfenicoles, betalactámicos, inhibidores de la vía del folato, tetraciclinas	XIII	1 (21)	*aadA2*		2015	(18)
China	Cerdo	Aminoglucósidos, anfenicoles, betalactámicos, inhibidores de la vía del folato, tetraciclinas	XIII	1 (2)	*aadA2; bla*_PSE-1_		2014-2017	(19)
Marruecos	Ganado vacuno	Aminoglucósidos, anfenicoles, betalactámicos, inhibidores de la vía del folato, tetraciclinas	XIII	5 (5)	*aadA2; bla*_PSE-1_		ND	(20)
Taiwán	Humano, cerdo, pollo, tortuga, paloma, pato, serpiente	Aminoglucósidos, anfenicoles, betalactámicos, inhibidores de la vía del folato, tetraciclinas	XIII	22 (191)	*aadA2; bla*_PSE-1_		ND	(20)
Taiwán	Humano, cerdo, pollo	Aminoglucósidos, anfenicoles, betalactámicos, inhibidores de la vía del folato, tetraciclinas	XIII	3 (191)	*drf12-orfFaadA2*		ND	(20)
Taiwán	Humano, cerdo	Aminoglucósidos, anfenicoles, betalactámicos, inhibidores de la vía del folato, tetraciclinas	XIII	1 (191)	*bla*_OXA-1_ *-aadA1*		ND	(20)
Taiwán	Humano, cerdo	Aminoglucósidos, anfenicoles, betalactámicos, inhibidores de la vía del folato, tetraciclinas	XIII	8 (191)	*aadB-catB3;* *blaOXA-1-aadA1*		ND	(20)
Reino Unido	Cerdo	Aminoglucósidos, anfenicoles, betalactámicos, inhibidores de la vía del folato, quinolonas, tetraciclinas	XII	2 (14)	*dfrA12-aadA2-* *cmlA1-aadA1*		2004-2006	(3)
Unión Europea	Cerdo	Aminoglucósidos, anfenicoles, betalactámicos, inhibidores de la vía del folato, quinolonas, tetraciclinas	XII	1 (43)	*bla*_OXA-1_ *-likeaadA1*		2004-2006	(4)
Unión Europea	Cerdo	Aminoglucósidos, anfenicoles, betalactámicos, inhibidores de la vía del folato, quinolonas, tetraciclinas	XII	2 (43)	*dfrA12-aadA2*		2004-2006	(4)
China	Carne preparada	Aminoglucósidos, anfenicoles, betalactámicos, inhibidores de la vía del folato, quinolonas, tetraciclinas	XII	1 (2)	*bla*_OXA-1_ *-aadA1*		2007-2008	(21)
España	Cerdo	Aminoglucósidos, anfenicoles, betalactámicos, inhibidores de la vía del folato, quinolonas, tetraciclinas	XII	2 (46)	*aadA2-bla*_PSE-1_		2008-2009	(9)
España	Cerdo	Aminoglucósidos, anfenicoles, betalactámicos, inhibidores de la vía del folato, quinolonas, tetraciclinas	XII	1 (46)	*aadA2; bla*_PSE-1_		2008-2009	(9)
España	Humano	Aminoglucósidos, anfenicoles, betalactámicos, inhibidores de la vía del folato, quinolonas, tetraciclinas	XII	11 (55)	*aadA2; bla*_PSE-1_		2009-2010	(10)
Irán	Humano, ave de corral	Aminoglucósidos, anfenicoles, betalactámicos, inhibidores de la vía del folato, quinolonas, tetraciclinas	XII	3 (4)	*aadA6-orfD*		2009-2010	(22)
Estados Unidos de América	Humano	Aminoglucósidos, anfenicoles, betalactámicos, inhibidores de la vía del folato, quinolonas, tetraciclinas	XII	3 (101) †	*aadA1, aadA2* *o aadA3-* *dfrA12*		2009-2014	(11)
Egipto	Carne de res, leche	Aminoglucósidos, anfenicoles, betalactámicos, inhibidores de la vía del folato, quinolonas, tetraciclinas	XII	3 (24)	*dfrA17-aadA5*		2010	(12)
Egipto	Carne de res	Aminoglucósidos, anfenicoles, betalactámicos, inhibidores de la vía del folato, quinolonas, tetraciclinas	XII	1 (24)	*dfrA1-aadA1*		2010	(12)
Egipto	Carne de res	Aminoglucósidos, anfenicoles, betalactámicos, inhibidores de la vía del folato, quinolonas, tetraciclinas	XII	2 (24)		*dfrA1-sat2-* *aadA1*	2010	(12)
Egipto	Carne de res, queso	Aminoglucósidos, anfenicoles, betalactámicos, inhibidores de la vía del folato, quinolonas, tetraciclinas	XII	5 (24)	*aadA2; bla*_PSE-1_		2010	(12)
Egipto	Leche	Aminoglucósidos, anfenicoles, betalactámicos, inhibidores de la vía del folato, quinolonas, tetraciclinas	XII	1 (24)	*dfrA15bcm1A4-* *aadA2*		2010	(12)
Egipto	Pollo	Aminoglucósidos, anfenicoles, betalactámicos, inhibidores de la vía del folato, quinolonas, tetraciclinas	XII	1 (6)	*aadA2; bla*_PSE-1_		2011	(13)
Egipto	Pollo	Aminoglucósidos, anfenicoles, betalactámicos, inhibidores de la vía del folato, quinolonas, tetraciclinas	XII	1 (6)	*dfrA17-aadA5*		2011	(13)
Egipto	Pollo	Aminoglucósidos, anfenicoles, betalactámicos, inhibidores de la vía del folato, quinolonas, tetraciclinas	XII	1 (6)	*aadA1*		2011	(13)
República Checa	Gaviota de cabeza negra (*Chroicocephalus* *ridibundus*)	Aminoglucósidos, anfenicoles, betalactámicos, inhibidores de la vía del folato, quinolonas, tetraciclinas	XII	1 (3)	*aadA2; bla*_PSE-1_		2012	(23)
China	Humano	Aminoglucósidos, anfenicoles, betalactámicos, inhibidores de la vía del folato, quinolonas, tetraciclinas	XII	27 (36)	*dhrA12-orfFaadA2*		2013-2015	(24)
China	Humano	Aminoglucósidos, anfenicoles, betalactámicos, inhibidores de la vía del folato, quinolonas, tetraciclinas	XII	5 (36)	*bla*_OXA-1_ *-* *aadA1*		2013-2015	(24)
China	Cerdo	Aminoglucósidos, anfenicoles, betalactámicos, inhibidores de la vía del folato, quinolonas, tetraciclinas	XII	4 (5)	*aadA2*		2016	(25)
Unión Europea	Cerdo	Aminoglucósidos, anfenicoles, betalactámicos, inhibidores de la vía del folato, quinolonas, tetraciclinas	XII	1 (43)	*aadA2*		2004-2006	(4)
Unión Europea	Cerdo	Aminoglucósidos, anfenicoles, betalactámicos, inhibidores de la vía del folato, quinolonas, tetraciclinas	XII	1 (43)	*aadA1-likeaadA2-* *dfrA12*		2004-2006	(4)
Egipto	Carne de res	Aminoglucósidos, anfenicoles, betalactámicos, inhibidores de la vía del folato, quinolonas, tetraciclinas	XII	1 (4)	*aadA2*		ND	(26)
Italia	Humano	Aminoglucósidos, betalactámicos, inhibidores de la vía del folato, tetraciclinas	XXVII	1 (14)	*dfrA1-aadA1*		2004-2006	(3)
Unión Europea	Cerdo	Aminoglucósidos, betalactámicos, inhibidores de la vía del folato, tetraciclinas	XXVII	2 (43)		*estX-sat2-* *aadA1*	2004-2006	(4)
España	Humano	Aminoglucósidos, betalactámicos, inhibidores de la vía del folato, tetraciclinas	XXVII	4 (55)	*dfrA1*		2009-2010	(10)
Egipto	Carne de res	Aminoglucósidos, betalactámicos, inhibidores de la vía del folato, tetraciclinas	XXVII	1 (24)	*dfrA12-orfaadA2*		2010	(12)
Egipto	Carne de res	Aminoglucósidos, betalactámicos, inhibidores de la vía del folato, tetraciclinas	XXVII	1 (24)	*dfrA15*		2010	(12)
Egipto	Leche	Aminoglucósidos, betalactámicos, inhibidores de la vía del folato, tetraciclinas	XXVII	1 (24)		*dfrA1-sat2*	2010	(12)
Brasil	Cerdo	Aminoglucósidos, betalactámicos, inhibidores de la vía del folato, quinolonas, tetraciclinas	XXVI	12 (45)	*dfrA12-orfFaadA27*		2007-2011	(7)
Egipto	Carne de res	Aminoglucósidos, betalactámicos, inhibidores de la vía del folato, quinolonas, tetraciclinas	XXVI	1 (24)	*dfrA12-orfaadA2*		2010	(12)
Egipto	Carne de res	Aminoglucósidos, betalactámicos, inhibidores de la vía del folato, quinolonas, tetraciclinas	XXVI	1 (24)	*dfrA1-aadA1*		2010	(12)
Egipto	Pollo	Aminoglucósidos, betalactámicos, inhibidores de la vía del folato, quinolonas, tetraciclinas	XXVI	1 (24)	*aadA2*		2010	(12)
China	Humano	Aminoglucósidos, betalactámicos, inhibidores de la vía del folato, quinolonas, tetraciclinas	XXVI	3 (36)	*dhrA12-orfFaadA2*		2013-2015	(24)
Egipto	Pollo	Aminoglucósidos, betalactámicos, inhibidores de la vía del folato, quinolonas, tetraciclinas	XXVI	3 (5)	*aadA7-aac(3)Id*		2016	(27)
China	Pato	Aminoglucósidos, betalactámicos, quinolonas, tetraciclinas	XXVIII	2 (24)	*aadA2*		2016	(28)
China	Gallinas	Aminoglucósidos, betalactámicos, quinolonas, tetraciclinas	XXVIII	1 (7)	*drfA1-aadA1*		2016	(29)
China	Human	Aminoglucósidos, betalactámicos, quinolonas, tetraciclinas	XXVIII	1 (1)	*drfA12-aadA2-* *aac(6’)-lb-crbla* *OXA-1-catB4-* *arr-3*		2017	(30)
Egipto	Pollo	Aminoglucósidos, betalactámicos, quinolonas, tetraciclinas	XXVIII	1 (1)	*sat; aac3-IdaadA7;* *aadA7*		2017-2018	(31)
China	Cordero	Aminoglucósidos, anfenicoles, betalactámicos, inhibidores de la vía del folato	III	1 (2)	*dfrA12-aadA2*		2007-2008	(21)
España	Humano	Aminoglucósidos, anfenicoles, betalactámicos, inhibidores de la vía del folato	III	1 (55)	*dfrA12-gcuFaadA2*		2009-2010	(10)
Egipto	Pollo	Aminoglucósidos, anfenicoles, betalactámicos, inhibidores de la vía del folato	III	1 (21)	*aadA1*		2015	(17)
Egipto	Pollo	Aminoglucósidos, anfenicoles, betalactámicos, inhibidores de la vía del folato	III	1 (21)	*aadA2*		2015	(17)
Egipto	Pollo	Aminoglucósidos, anfenicoles, betalactámicos, inhibidores de la vía del folato, macrólidos, quinolonas, tetraciclinas	V	1 (7)	*estX-sat*		2014-2015	(32)
Egipto	Humano	Aminoglucósidos, anfenicoles, betalactámicos, inhibidores de la vía del folato, macrólidos, quinolonas, tetraciclinas	V	1 (7)	*aadA4; dfrA15*		2014-2015	(32)
Egipto	Pollo	Aminoglucósidos, anfenicoles, betalactámicos, inhibidores de la vía del folato, macrólidos, quinolonas, tetraciclinas	V	1 (7)	*aadA2-lnuF*		2014-2015	(32)
Egipto	Pollo	Aminoglucósidos, anfenicoles, betalactámicos, inhibidores de la vía del folato, macrólidos, quinolonas, tetraciclinas	V	1 (7)	*aac(3)-IdaadA7*		2014-2015	(32)
Unión Europea	Cerdo	Aminoglucósidos, inhibidores de la vía del folato, tetraciclinas	XXXI	1 (43)	*aadA1*		2004-2006	(4)
Unión Europea	Cerdo	Aminoglucósidos, inhibidores de la vía del folato, tetraciclinas	XXXI	1 (43)	*aadA2*		2004-2006	(4)
Unión Europea	Cerdo	Aminoglucósidos, inhibidores de la vía del folato, tetraciclinas	XXXI	1 (43)	*aadA1-likeaadA2-* *dfrA12*		2004-2006	(4)
Estados Unidos de América	Humano, cerdo, bovino, ave de corral	Aminoglucósidos, inhibidores de la vía del folato, tetraciclinas	XXXI	24 (101) †	*aadA1, aadA2* *o aadA3*		2009-2014	(11)
Unión Europea	Cerdo	Aminoglucósidos, anfenicoles, inhibidores de la vía del folato, tetraciclinas	XIX	1 (43)	*aadA1*		2004-2006	(4)
Brasil	Cerdo	Aminoglucósidos, anfenicoles, inhibidores de la vía del folato, tetraciclinas	XIX	1 (45)	*aadA23*		2007-2011	(7)
Egipto	Pollo	Aminoglucósidos, anfenicoles, inhibidores de la vía del folato, tetraciclinas	XIX	1 (21)	*dfrA15; aadA2*		2015	(17)
Italia	Cerdo	Aminoglucósidos, anfenicoles, betalactámicos, inhibidores de la vía del folato, nitrofuranos, tetraciclinas	X	1 (14)	*dfrA12-aadA2-* *cmlA1-aadA1*		2004-2006	(3)
Italia	Humano	Aminoglucósidos, anfenicoles, betalactámicos, inhibidores de la vía del folato, nitrofuranos, tetraciclinas	X	2 (14)	*blaOXA-1-aadA1*		2004-2006	(3)
Reino Unido	Cerdo	Aminoglucósidos, anfenicoles, betalactámicos, inhibidores de la vía del folato, nitrofuranos, tetraciclinas	X	1 (14)	*dfrA12-aadA2-* *cmlA1-aadA1*		2004-2006	(3)
Irán	Humano	Aminoglucósidos, anfenicoles, betalactámicos, inhibidores de la vía del folato, quinolonas	XI	1 (3)		*dfrA14- lsp*	2008-2009	(33)
Egipto	Pollo	Aminoglucósidos, anfenicoles, betalactámicos, inhibidores de la vía del folato, quinolonas	XI	1 (24)	*aadB-catB3*		2010	(12)
Corea	Cerdo	Aminoglucósidos, anfenicoles, betalactámicos, inhibidores de la vía del folato, quinolonas	XI	1 (6)	*dfrA12-aadA2*		2016-2017	(34)
Egipto	Pollo	Aminoglucósidos, anfenicoles, betalactámicos, tetraciclinas	XVI	1 (21)	*dfrA15; aadA2*		2015	(17)
Egipto	Pollo	Aminoglucósidos, anfenicoles, betalactámicos, tetraciclinas	XVI	1 (21)	*dfrA15; aadA2;* *aadA1; dfrA12-* *orfF-aadA27*		2015	(17)
Corea	Cerdo	Aminoglucósidos, anfenicoles, betalactámicos, tetraciclinas	XVI	1 (6)	*aadA2-blaPSE-1*		2016-2017	(34)
Egipto	Pollo	Aminoglucósidos, anfenicoles, betalactámicos, inhibidores de la vía del folato, macrólidos, rifamicinas	VI	1 (11)	*dfrA12-orfFaadA27;* *aadA23; dfrA15*		2015-2016	(35)
Egipto	Pollo	Aminoglucósidos, anfenicoles, betalactámicos, inhibidores de la vía del folato, macrólidos, rifamicinas	VI	1 (11)		*dfrA1-sat2-* *aadA30*	2015-2016	(35)
España	Humano	Aminoglucósidos, betalactámicos, inhibidores de la vía del folato	XXI	2 (5)	*bla*_OXA-1_ *-aadA1*		2002-2004	(1)
Unión Europea	Cerdo	Aminoglucósidos, betalactámicos, inhibidores de la vía del folato	XXI	1 (43)	*aadA2; bla*_PSE-1_		2004-2006	(4)
Tailandia	Cerdo	Aminoglucósidos, betalactámicos, tetraciclinas	XXIX	1 (11) ‡	*aadA1*		2011-2013	(36)
Corea	Cerdo	Aminoglucósidos, betalactámicos, tetraciclinas	XXIX	1 (6)	*dfrA12-aadA2*		2016-2017	2016-2017 (34)
España	Humano	Anfenicoles, betalactámicos, inhibidores de la vía del folato, tetraciclinas	XXXVII	4 (55)	*bla*_OXA-1_ *-aadA1*		2009-2010	(10)
República Checa	Planta de tratamiento de aguas residuales	Anfenicoles, betalactámicos, inhibidores de la vía del folato, tetraciclinas	XXXVII	1 (3)	*aadA2; bla*_PSE-1_		2012	(23)
Egipto	Pollo	Aminoglucósidos, anfenicoles, betalactámicos	I	1 (21)	*dfrA15; aadA2*		2015	(17)
Unión Europea	Cerdo	Aminoglucósidos, anfenicoles, betalactámicos, fosfonatos, inhibidores de la vía del folato, quinolonas, tetraciclinas	II	1 (43)	*aadA2; bla*_PSE-1_		2004-2006	(4)
Egipto	Pollo	Aminoglucósidos, anfenicoles, betalactámicos, inhibidores de la vía del folato, macrólidos, quinolonas, rifamicinas, tetraciclinas	IV	1 (11)		*dfrA1-sat2-* *aadA30*	2015-2016	(35)
Egipto	Pollo	Aminoglucósidos, anfenicoles, betalactámicos, inhibidores de la vía del folato, macrólidos, rifamicinas, tetraciclinas	VII	2 (11)		*dfrA1-sat2-* *aadA30*	2015-2016	(35)
Reino Unido	Cerdo	Aminoglucósidos, anfenicoles, betalactámicos, inhibidores de la vía del folato, nitrofuranos	VIII	1 (14)	*dfrA12-aadA2-* *cmlA1-aadA1*		2004-2006	(3)
China	Cerdo	Aminoglucósidos, anfenicoles, betalactámicos, inhibidores de la vía del folato, nitrofuranos, quinolonas, tetraciclinas	IX	1 (2)	*dfrA12-aadA21*		2014-2017	(18)
China	Pollo	Aminoglucósidos, anfenicoles, betalactámicos, polimixinas, quinolonas, tetraciclinas	XIV	1 (5)	*aadA5-blaOXA*		2018	(37)
China	Cerdo	Aminoglucósidos, anfenicoles, betalactámicos, quinolonas, tetraciclinas	XV	1 (24)	*aadA2*		2016	(28)
Egipto	Humano	Aminoglucósidos, anfenicoles, inhibidores de la vìa del folato	XVII	1 (21)	*dfrA15; aadA2*		2015	(17)
India	Humano, ambiental (ave, cabra, ganado)	Aminoglucósidos, anfenicoles, inhibidores de la vía del folato, quinolonas, tetraciclinas	XVIII	9 (9)	*dfrA12-orfFaadA2-* *cmlA1-* *aadA1*		2010-2017	(38)
Unión Europea	Cerdo	Aminoglucósidos, betalactámicos, fosfonatos, inhibidores de la vía del folato, quinolonas	XX	1 (43)	*bla*_OXA-1_ *-likeaadA1*		2004-2006	(4)
Egipto	Pollo	Aminoglucósidos, betalactámicos, inhibidores de la vía del folato, macrólidos, quinolonas, tetraciclinas	XXII	1 (7)	*sat; aac(3)-IdaadA7*		2014-2015	(32)
Egipto	Pollo	Aminoglucósidos, betalactámicos, inhibidores de la vía del folato, macrólidos, rifamicinas, tetraciclinas	XXIII	1 (11)		*dfrA1-sat2-* *aadA30*	2015-2016	(35)
Unión Europea	Cerdo	Aminoglucósidos, betalactámicos, inhibidores de la vía del folato, polimixinas, tetraciclinas	XXIV	1 (43)	*aadA1-likedfrA1-* *like-aac3*		2004-2006	(4)
Corea	Cerdo	Aminoglucósidos, betalactámicos, inhibidores de la vía del folato, quinolonas	XXV	1 (6)	*dfrA12-aadA2*		2016-2017	(34)
Irán	Humano	Aminoglucósidos, inhibidores de la vía del folato, quinolonas, tetraciclinas	XXX	1 (4)	*aadA2*		2009-2010	(22)
China	Pollo	Aminoglucósidos, polimixinas, quinolonas, tetraciclinas	XXXII	1 (5)	*dfrA17-aadA5*		2018	(37)
Egipto	Humano	Anfenicoles, betalactámicos, inhibidores de la vía del folato	XXXIII	1 (21)	*dfrA15; aadA2;* *aadA1; dfrA12-* *orfF-aadA27*		2015	(17)
Egipto	Pollo	Anfenicoles, betalactámicos, inhibidores de la vía del folato, macrólidos, rifamicinas	XXXIV	1 (11)		*dfrA1-sat2-* *aadA30,* *sat2-aadA1,* *catB2*	2015-2016	(35)
Egipto	Ave de corral	Anfenicoles, betalactámicos, inhibidores de la vía del folato, quinolonas	XXXV	1 (4)	*dfrA15-dfrA17*		ND	(26)
Egipto	Pollo	Anfenicoles, betalactámicos, inhibidores de la vía del folato, rifamicinas	XXXVI	1 (11)		*dfrA1-sat2-* *aadA30,* *catB2*	2015-2016	(35)
Corea	Cerdo	Anfenicoles, betalactámicos, tetraciclinas	XXXVIII	2 (6)	*aadA2-bla*_PSE-1_		2016-2017	(34)
Egipto	Pollo	Anfenicoles, inhibidores de la vía del folato, tetraciclinas	XXXIX	1 (21)	*dfrA15*		2015	(17)
China	Pollo	Betalactámicos, polimixinas, quinolonas, tetraciclinas	XL	1 (5)	*dfrA1-aadA1*		2018	(37)
Unión Europea	Cerdo	Aminoglucósidos, anfenicoles, inhibidores de la vía del folato, quinolonas	XLI	1 (43)	*aadA2*		2004-2006	(4)
España	Humano	Betalactámicos, inhibidores de la vía del folato, tetraciclinas	XLII	1 (55)	*blaOXA-1-aadA1*		2009-2010	(10)

Int: integrón; Int1: integrón de clase 1; Int2: integrón de clase 2; RV: región variable; ND: no disponible

* Otros tres aislamientos MDR fueron positivos para integrones de clase 1 de 400 pb, pero no se describen los genes de la región variable.

† Se realizó conteo de aislamientos MDR con y sin integrones: se consideró MDR a un aislamiento resistente a 3 o más familias de antibióticos, los aislamientos con resistencia intermedia fueron considerados resistentes al antibiótico probado.

‡ Número total de aislamientos de *S.* Typhimurium, el documento no especifica si todos son MDR.

### Referencias

1. Herrero-Fresno A, Rodicio R, Montero I, García P, Rodicio MR. Transposition and homologous recombination drive evolution of pUO-StVR2, a multidrug resistance derivative of pSLT, the virulence plasmid specific of *Salmonella enterica* serovar Typhimurium. Infect Genet Evol. 2015;29:99-102. https://doi.org/10.1016/j.meegid.2014.11.010

2. Montero I, Herrero A, Mendoza MC, Rodicio R, Rodicio MR. Virulence-resistance plasmids (pUO-StVR2-like) in meat isolates of *Salmonella enterica* serovar Typhimurium. Food Res Int. 2012;45:1025-9. https://doi.org/10.1016/j.foodres.2011.04.014

3. Beutlich J, Rodicio MR, Mendoza MC, García P, Kirchner M, Luzzi I, *et al. Salmonella enterica* serovar Typhimurium virulence-resistance plasmids derived from the pSLT carrying nonconventional class 1 integrons with dfrA12 gene in their variable region and sul3 in the 3’ conserved segment. Microb Drug Resist. 2013;19:437-45. https://doi.org/10.1089/mdr.2012.0226

4. Argüello H, Guerra B, Rodríguez I, Rubio P, Carvajal A. Characterization of antimicrobial resistance determinants and class 1 and class 2 integrons in *Salmonella enterica* spp. multidrug-resistant isolates from pigs. Genes (Basel). 2018;9. https://doi.org/10.3390/genes9050256

5. De Vito D, Monno R, Nuccio F, Legretto M, Oliva M, Coscia MF, *et al.* Diffusion and persistence of multidrug resistant *Salmonella* Typhimurium strains phage type DT120 in southern Italy. Biomed Res Int. 2015;2015:265042. https://doi.org/10.1155/2015/265042

6. Gomes-Neves E, Antunes P, Manageiro V, Gärtner F, Caniça M, da Costa JMC, *et al.* Clinically relevant multidrug resistant *Salmonella enterica* in swine and meat handlers at the abattoir. Vet Microbiol. 2014;168:229-33. https://doi.org/10.1016/j.vetmic.2013.10.017

7. Lopes GV, Michael GB, Cardoso M, Schwarz S. Antimicrobial resistance and class 1 integron-associated gene cassettes in *Salmonella enterica* serovar Typhimurium isolated from pigs at slaughter and abattoir environment. Vet Microbiol. 2016;194:84-92. https://doi.org/10.1016/j.vetmic.2016.04.020

8. Oliva M, Calia C, Ferrara M, D’Addabbo P, Scrascia M, Mulè G, *et al.* Antimicrobial resistance gene shuffling and a three-element mobilisation system in the monophasic Salmonella Typhimurium strain ST1030. Plasmid. 2020;111:102532. https://doi.org/10.1016/j.plasmid.2020.102532

9. Garrido V, Sánchez S, San Román B, Zabalza-Baranguá A, Díaz-Tendero Y, de Frutos C, *et al.* Simultaneous infections by different *Salmonella* strains in mesenteric lymph nodes of finishing pigs. BMC Vet Res. 2014;10:59. https://doi.org/10.1186/1746-6148-10-59

10. de Toro M, Seral C, Rojo-Bezares B, Torres C, Castillo FJ, Sáenz Y. Antibiotic resistance and virulence factors in clinical *Salmonella enterica* isolates. Enferm Infecc Microbiol Clin. 2014;32:4-10. https://doi.org/10.1016/j.eimc.2013.03.006

11. Rao S, Linke L, Doster E, Hyatt D, Burgess BA, Magnuson R, *et al.* Genomic diversity of class I integrons from antimicrobial resistant strains of *Salmonella* Typhimurium isolated from livestock, poultry and humans. PLoS ONE. 2020;15:e0243477. https://doi.org/10.1371/journal.pone.0243477

12. Ahmed AM, Shimamoto T, Shimamoto T. Characterization of integrons and resistance genes in multidrug-resistant *Salmonella enterica* isolated from meat and dairy products in Egypt. Int J Food Microbiol. 2014;189:39-44. https://doi.org/10.1016/j.ijfoodmicro.2014.07.031

13. Ahmed AM, Shimamoto T. Genetic analysis of multiple antimicrobial resistance in *Salmonella* isolated from diseased broilers in Egypt. Microbiol Immunol. 2012;56:254-61. https://doi.org/10.1111/j.1348-0421.2012.00429.x

14. Campos J, Mourão J, Marçal S, Machado J, Novais C, Peixe L, *et al.* Clinical *Salmonella* Typhimurium ST34 with metal tolerance genes and an IncHI2 plasmid carrying *oqxABaac( 6')-Ib-cr* from Europe. J Antimicrob Chemother. 2016;71:843-5. https://doi.org/10.1093/jac/dkv409

15. Monte DFM, Sellera FP, Lopes R, Keelara S, Landgraf M, Greene S, *et al*. Class 1 integronborne cassettes harboring blaCARB-2 gene in multidrug-resistant and virulent *Salmonella* Typhimurium ST19 strains recovered from clinical human stool samples, United States. PLoS ONE. 2020;15:e0240978. https://doi.org/10.1371/journal.pone.0240978

16. El-Sharkawy H, Tahoun A, El-Gohary AE-GA, El-Abasy M, El-Khayat F, Gillespie T, *et al.* Epidemiological, molecular characterization and antibiotic resistance of *Salmonella* entérica serovars isolated from chicken farms in Egypt. Gut Pathog. 2017;9:8. https://doi.org/10.1186/s13099-017-0157-1

17. Ahmed HA, El-Hofy FI, Shafik SM, Abdelrahman MA, Elsaid GA. Characterization of virulence-associated genes, antimicrobial resistance genes, and class 1 integrons in *Salmonella enterica* serovar Typhimurium isolates from chicken meat and humans in Egypt. Foodborne Pathog Dis. 2016;13:281-8. https://doi.org/10.1089/fpd.2015.2097

18. Zhou M, Li X, Hou W, Wang H, Paoli GC, Shi X. Incidence and characterization of salmonella isolates from raw meat products sold at small markets in Hubei province, China. Front Microbiol. 2019;10:2265. https://doi.org/10.3389/fmicb.2019.02265

19. Murgia M, Bouchrif B, Timinouni M, Al-Qahtani A, Al-Ahdal MN, Cappuccinelli P, *et al.* Antibiotic resistance determinants and genetic analysis of *Salmonella enterica* isolated from food in Morocco. Int J Food Microbiol. 2015;215:31-9. https://doi.org/10.1016/j.ijfoodmicro.2015.08.003

20. Hsu Y-M, Tang C-Y, Lin H, Chen Y-H, Chen Y-L, Su Y-H, *et al.* Comparative study of class 1 integron, ampicillin, chloramphenicol, streptomycin, sulfamethoxazole, tetracycline (ACSSuT) and fluoroquinolone resistance in various *Salmonella* serovars from humans and animals. Comp Immunol Microbiol Infect Dis. 2013;36:9-16. https://doi.org/10.1016/j.cimid.2012.08.004

21. Yu T, Jiang X, Zhou Q, Wu J, Wu Z. Antimicrobial resistance, class 1 integrons, and horizontal transfer in *Salmonella* isolated from retail food in Henan, China. J Infect Dev Ctries. 2014;8:705-11. https://doi.org/10.3855/jidc.4190

22. Firoozeh F, Zahraei-Salehi T, Shahcheraghi F. Molecular clonality and detection of class 1 integron in multidrug-resistant *Salmonella enterica* isolates from animal and human in Iran. Microb Drug Resist. 2014;20:517-24. https://doi.org/10.1089/mdr.2013.0198

23. Masarikova M, Manga I, Cizek A, Dolejska M, Oravcova V, Myskova P, *et al*. *Salmonella enterica* resistant to antimicrobials in wastewater effluents and black-headed gulls in the Czech Republic, 2012. Sci Total Environ. 2016;542:102-7. https://doi.org/10.1016/j.scitotenv.2015.10.069

24. Yuan J, Guo W. Mechanisms of resistance to quinolones in *Salmonella* Typhimurium from patients with infectious diarrhea. Microbiol Immunol. 2017;61:138-43. https://doi.org/10.1111/1348-0421.12476

25. Zhao X, Ye C, Chang W, Sun S. Serotype distribution, antimicrobial resistance, and class 1 integrons profiles of *Salmonella* from animals in slaughterhouses in Shandong Province, China. Front Microbiol. 2017;8:1049. https://doi.org/10.3389/fmicb.2017.01049

26. Moawad AA, Hotzel H, Awad O, Tomaso H, Neubauer H, Hafez HM, *et al.* Occurrence of *Salmonella enterica* and *Escherichia coli* in raw chicken and beef meat in northern Egypt and dissemination of their antibiotic resistance markers. Gut Pathog. 2017;9:57. https://doi.org/10.1186/s13099-017-0206-9

27. Elkenany RM, Eladl AH, El-Shafei RA. Genetic characterisation of class 1 integrons among multidrug-resistant *Salmonella* serotypes in broiler chicken farms. J Glob Antimicrob Resist. 2018;14:202-8. https://doi.org/10.1016/j.jgar.2018.04.009

28. Zhao X, Yang J, Zhang B, Sun S, Chang W. Characterization of integrons and resistance genes in *Salmonella* isolates from farm animals in Shandong Province, China. Front Microbiol. 2017;8:1300. https://doi.org/10.3389/fmicb.2017.01300

29. Li S, Zhou Y, Miao Z. Prevalence and antibiotic resistance of non-typhoidal *Salmonella* Isolated from raw chicken carcasses of commercial broilers and spent hens in Tai’an, China. Front Microbiol. 2017;8:2106. https://doi.org/10.3389/fmicb.2017.02106

30. Yao L, Ding Y, Ding M, Yan X, Zhang F, Zhang Z, *et al.* Characterization of a novel class 1 integron InSW39 and a novel transposon Tn5393k identified in an imipenem-nonsusceptible *Salmonella* Typhimurium strain in Sichuan, China. Diagn Microbiol Infect Dis. 2020;99:115263. https://doi.org/10.1016/j.diagmicrobio.2020.115263

31. Shabana S, Helmy S, Hegazy AE-H. Characterization of class 1 integrons and some anti-microbial resistance genes in *Salmonella* species isolated from poultry in Egypt. SVR. 2019;56(22-Suppl.). https://doi.org/10.26873/SVR-813-2019

32. Gharieb RM, Tartor YH, Khedr MHE. Non-typhoidal Salmonella in poultry meat and diarrhoeic patients: Prevalence, antibiogram, virulotyping, molecular detection and sequencing of class I integrons in multidrug resistant strains. Gut Pathog. 2015;7:34. https://doi.org/10.1186/s13099-015-0081-1

33. Rajaei B, Rad NS, Badmasti F, Razavi MR, Aghasadeghi MR, Saboohi R, *et al.* Molecular detection of antimicrobial resistance gene cassettes associated with class 2 integron in *Salmonella* serovars isolated in Iran. BMRJ. 2014;4:132-41. https://doi.org/10.9734/BMRJ/2014/4639

34. Noh EB, Kim YB, Jeon HY, Seo KW, Son SH, Lee YJ. Antimicrobial resistance and genetic diversity of *Salmonella* serotypes recovered from edible pork offal from Korea. Microb Drug Resist. 2019;25:1514-20. https://doi.org/10.1089/mdr.2019.0010

35. El-Demerdash AS, Aggour MG, El-Azzouny MM, Abou-Khadra SH. Molecular analysis of integron gene cassette arrays associated multi-drug resistant Enterobacteriaceae isolates from poultry. Cell Mol Biol (Noisy-le-grand). 2018;64:149-56. https://doi.org/10.14715/cmb/2018.64.5.25

36. Boonkhot P, Tadee P, Yamsakul P, Pocharoen C, Chokesajjawatee N, Patchanee P. Class 1 integrons characterization and multilocus sequence typing of *Salmonella* spp. From swine production chains in Chiang Mai and Lamphun provinces, Thailand. Jpn J Vet Res. 2015;63:83-94.

37. Zhao X, Hu M, Zhang Q, Zhao C, Zhang Y, Li L, *et al.* Characterization of integrons and antimicrobial resistance in *Salmonella* from broilers in Shandong, China. Poult Sci. 2020;99:7046-54. https://doi.org/10.1016/j.psj.2020.09.071

38. Jain P, Sudhanthirakodi S, Chowdhury G, Joshi S, Anandan S, Ray U, *et al.* Antimicrobial resistance, plasmid, virulence, multilocus sequence typing and pulsed-field gel electrophoresis profiles of *Salmonella enterica* serovar Typhimurium clinical and environmental isolates from India. PLoS ONE. 2018;13:e0207954. https://doi.org/10.1371/journal.pone.0207954
